# “Serious skin & soft tissue infections in rheumatoid arthritis patients taking anti-tumor necrosis factor alpha drugs: a nested case–control study”

**DOI:** 10.1186/1471-2334-13-533

**Published:** 2013-11-11

**Authors:** Ngoc J Wasson, Cara D Varley, Pascal Schwab, Rongwei Fu, Kevin L Winthrop

**Affiliations:** 1 Oregon Health and Sciences University, Portland, Oregon, USA; 2 Portland VA Medical Center, Portland, Oregon, USA

**Keywords:** Infectious skin diseases, Tumor necrosis factor-alpha, Anti-TNF therapy, Arthritis, Rheumatoid

## Abstract

**Background:**

Anti-tumor necrosis factor alpha (anti-TNF) drugs are very effective for the treatment of rheumatoid arthritis but may increase the risk of serious bacterial infections. We assessed the association between the risk of serious skin and soft tissue infections (SSSTI) and the use of these agents in rheumatoid arthritis patients (RA).

**Methods:**

We conducted a nested case–control study among rheumatoid arthritis patients in the Veterans Integrated Service Network 20 from 2000–2008. We identified rheumatoid arthritis patients with SSSTI, matched them to three sets of RA controls and used conditional logistic regression to compare the risk of SSSTI between patients treated and those not treated with an anti-TNF drug, after adjusting for known confounders and important covariates. Limited by the design, we could not assess (absolute) risk but only relative risk in terms of association.

**Results:**

Among the 97 cases and 291 controls, 90 percent were male, 62 percent white, with a mean age of 63 years. Twenty percent received anti-TNF drugs during the study period. Thirty-nine percent of cases and 15 percent of controls died, (OR 3.5, 95% CI: 2.033, 6.11, p <0.01). Diabetes mellitus (37%), kidney disease (16%) and a history of skin infections (27%) were common among cases. Based on conditional logistic regression, anti-TNF use was not significantly associated with skin and soft tissue infections (OR 1.1, 95% CI: 0.61-2.03, p = 0.92). However, patients with diabetes mellitus (OR 2.5, 95% CI: 1.53-4.13, p = 0.01) or a prior history of skin infection (OR 5.7, 95% CI: 2.87-11.43, p <0.01) were more likely to have skin and soft tissue infections.

**Conclusion:**

Use of anti-TNF therapy among RA patients was not associated with an increased risk of SSSTI, but patients with diabetes mellitus and those with a history of prior skin infection were significantly more likely to have SSSTI and mortality was higher among cases than controls in this veteran cohort.

## Background

More than 2.5 million patients in the U.S. have rheumatoid arthritis (RA), [1] and world wide the prevalence is estimated to be 1% of the population [2]. RA is a chronic inflammatory symmetrical polyarthritis and if untreated results in joint erosions, joint deformities and significant disability. Extra-articular manifestations including subcutaneous nodules, pulmonary manifestations, vasculitis, and inflammatory eye disease may be particularly challenging to treat. Early diagnosis and recent advances in RA treatment may prevent disease progression and improve long-term outcomes [3]. RA patients have an increased lifetime risk for cardiovascular disease [4] and are at greater risk of developing infections [5-7]. The problem of increased infections and higher rates of mortality from infections in RA patients compared to individuals without RA has been well described in the past decade. A pivotal cohort study evaluating RA patient data for up to 15 years found a twofold greater risk of infection in patients with RA compared to those without RA, in particular serious infections of the lung, skin, bone, and joint [8]. Specific causes for the high rate of infections remain indistinct but having compromised immune systems may put this cohort at substantial risk for severe complications [9].

### Increased risk of skin and soft tissue infections in RA patients

Skin and soft tissue infections (SSSTI) represent an important cause of morbidity for RA patients [9].

Among the increased infections noted, SSSTI are a leading cause of hospitalization and antibiotic drug use [10-13]. A 2009 study to assess the prevalence of serious infections among RA patients receiving anti-TNF therapy found that 20.5 percent of the serious infections identified were SSSTI [14].

SSSTI are difficult to treat in immosuppressed patients, often become life threatening, and represent a substantial public health burden [15,16]. The increase of SSSTI and continuing escalation in methicillin-resistant staphylococcus aureus rates andother serious infections raises the possibility that increased infections among RA patients and other autoimmune disease populations could be related to treatment with immunosuppressive agents.

### Effect of immunosuppressive drugs on the risk of infection in RA patients

A wide variety of drugs are used to minimize inflammation, treat pain, and slow the progression of RA. Treatments range from mild to aggressive therapies depending on the severity of disease activity. Therapies for RA include nonsteroidal anti-inflammatory drugs (NSAIDs), corticosteroids, disease-modifying antirheumatic drugs (DMARDs), and biologic agents including anti-TNF agents (drugs that inhibit tumor necrosis factor-apha).

Nearly half of the RA patients in the United States have received anti-TNF drugs [17]. Although anti-TNF drug treatment is highly successful, studies conducted in the past ten years indicate that the risk of developing a serious infection is higher among patients taking these drugs, potentially because of the resultant immunosuppression [6,18].

### Does anti-TNF use in RA patients increase the risk of infection?

It remains unclear how immunosuppressive therapies might influence the risk of SSSTI [8]. Numerous studies have assessed the increased risk of infection among patients receiving anti- TNF drug therapy [16]. A small systematic review (9 trials) [19] and a lager meta-analysis [20], both found an association between anti-TNF drug treatment and an increase in all serious infections. In two large retrospective cohort studies, including one conducted among U.S. Veterans Administration Hospitals patients, [21] one found a slightly increased risk (HR 1.24) between TNFi and hospitalized infection but it was of less magnitude than that for prednisone and the other study found no association [22].

Despite these studies, research into the increased rate of serious infections in RA patients since the introduction of anti-TNF therapy in the U.S. is still limited [19]. Results across studies are inconsistent, establishing a need for further research to identify potential risk factors and better assess the safety of anti-TNF therapy. The increasing trend in anti-TNF drug use to treat RA makes investigating the association of biologics with serious skin and soft tissue infections a clinically and epidemiologically important endeavor.

The high rate of mortality and co-morbidities among patients in the Veterans Integrated Service Network (VISN) makes understanding risk factors for serious infections of particular significance for this at risk population, yet there remains a scarcity of research specifically investigating the association of SSSTIs in relation to anti-TNF therapy in this cohort.

The objective of this study was to determine if anti-TNF therapy is associated with an increased occurrence of serious skin and soft tissue infections requiring hospitalization in patients with rheumatoid arthritis. Relatively little VA specific data has been published on this topic and that this is a unique population quite distinct from other RA populations with regard to sex, socioeconomic class, and other factors.

## Methods

### Study design and population

We conducted a nested case–control study among RA patients in the VISN 20 from 2000–2008 to evaluate anti-TNF drug use and SSSTI requiring hospitalization adjusting for known confounders and clinically important covariates such as age, sex, ethnicity, other immunosuppressant drug use, co-morbidities, and other autoimmune diseases. This study was approved by the Portland VA Medical Center Institutional Review Board.

### Patient cohort definition

#### RA cohort definition

The cohort for this study was comprised of patients in the United States Veterans Integrated Service Network 20, consisting of individuals who lived predominantly in Washington, Oregon, and Idaho. We defined RA patients as patients having at least one international classification of disease (ICD-9) code for RA (714), documentation of a rheumatology visit two or more times, and at least one prescription fill for a disease-modifying anti-rheumatic therapy (DMARD) during the study time-period (verified by pharmacy codes indicating at least one prescription fill for at least one drug used to treat RA).

### Definition of terms and variables

#### Hospitalized SSSTI case finding and control selection

Cases and controls were derived from comparable Veterans Administration (VA) populations Similar to prior studies, we used frequency of rheumatology visits in the 12 months prior to index date as a proxy for disease. We identified cases of SSSTI as patients having an inpatient SSSTI discharge codes validated to have high positive predictive value for disease [23,24] and enrolled all cases meeting the predefined criteria. For each case, we assigned an index date corresponding to the date of the hospital discharge. The cases were matched with controls identified as RA patients with an outpatient physician service date within +/− 30 days of each case’s index date at the same VA facility as the case’s SSSTI hospitalization. Three sets of controls meeting this definition were randomly matched to each case.

#### Drug exposure determination and covariate measures

We considered cases and controls as exposed to anti-TNF agents if they filled a prescription for etanercept or adalimumab or received an infusion for infliximab in the 90 days before the index date. To ascertain potential confounders, we collected the following covariates for each case and control, (defined by presence of disease specific inpatient or outpatient ICD-9 codes present prior to the index date during the study time-period): a history of diabetes mellitus, chronic kidney disease, neoplasm, chronic bronchitis, gastro esophageal reflux disease, human immunodeficiency virus infection, chronic liver disease, Crohn’s disease, ulcerative colitis, ankylosing spondylitis, uveitis, psoriatic arthritis and prior SSSTI (ICD-9 record of SSSTI anytime prior to study period) and a history of prior hospitalization for infections. Concomitant medications assessed included use of methotrexate, prednisone, azathioprine, leflunomide, hydroxychlorquine, and sulfasalazine within 30 days of the index date as well as prednisone use in the six months prior to the index date and antibiotic use 90 days prior to the index date. Medication use was defined by at least one record of pharmacy fill during the study period within the specified time frames for each medication.

### Statistical analysis

We compared differences in covariates among cases and controls using Fisher’s exact tests for categorical variables, and two-sample t-tests for continuous variables. We used conditional logistic regression to evaluate the association of SSSTI with covariates and specifically immunosuppressive drug use. Variables with a univariate association with SSSTI with a *p*-value ≤ 0.25 and variables identified a priori as clinically and epidemiologically important independent covariates were considered for building a conditional logistic regression multivariate model [25]. We performed step-wise backward elimination and included immunosuppressive use and all other variables with α ≤ 0.05 in our final model. Statistical analyses were conducted using SAS version 9.2 (SAS Institute Inc., Cary, N.C. 2008).

## Results

We identified 97 cases and 291 controls from the entire cohort of 6,305 RA patients in VISN 20 during the study period. Cases and controls were similar with regard to key prognostic factors for disease severity and did not differ significantly with regard to age, race, and gender or other demographic characteristics. Most patients were male (92% cases and 89% controls) and white (77% cases and 67% controls); with a mean age of 63 years for cases and 61 years for controls (p > 0.05 for all baseline comparisons). Ninety-five percent of cases and 87 percent of controls were 50 years old or greater. The average number of rheumatology clinical visits was similar for cases and controls; 35 percent of cases and 41 percent of controls had greater than 15 rheumatology clinical visits in the 12 months prior to the index date suggesting cases and controls had comparable RA disease activity.

Over the entire study time period 30 percent of cases and 24 percent of controls were prescribed anti-TNF drugs at least one time prior to the case’s index date. Diabetes mellitus (37%), kidney disease (16%) and having a prior history of skin infections (27%) were common among cases, but not among controls (18%, 4%, and 5% respectively).

Results of the univariate analysis of risk factors associated with developing SSSTI are presented in Table 1. Based on univariate comparison of cases and controls, diabetes mellitus, chronic kidney disease, chronic bronchitis, prior history of skin infection, pneumonia, discharge 3 months prior, prednisone use within 30 days of the index date and antibiotic use 90 days prior to the index date were considered for multivariate model. Age and race were also included in the initial multivariate model to assess potential confounding.

**Table 1:** Univariate analysis of covariates including patient drug exposures, comorbidities, and the association with hospitalized SSSTI

**Variable**	**Case**	**Control**	**Odds Ratio**	**95%**	**P-Value**
	**N = 97**	**N = 291**		**Confidence Interval**	
	**Frequency (Percent)**	**Frequency (Percent)**			
**MEDICATONS**					
**Anti-TNF Drugs**					
Biologics 90 days before index date	19 (19.59)	53 (18.15)	1.107	0.604, 2.028	0.74
Biologics Anytime Before Index	29 (29.90)	70 (23.97)	1.388	0.815, 2.363	0.23
ETANERCEPT Anytime Before Index	16 (16.49)	42 (14.38)	1.184	0.631, 2.22	0.60
INFLIXIMAB IV Anytime Before Index	3 (3.09)	13 (4.45)	0.857	0.178, 4.126	0.85
ADALIMUMAB Anytime Before Index	1 (1.03)	7 (2.40)	0.404	0.47, 3.462	0.41
Monoclonals Only: INFLIXIMAB/ ADALIMUMAB	7 (7.22)	14 (4.79)	0.633	0.178, 2.245	0.48
**Concomitant Medications**					
Methotrexate within 30 days of index date	20 (20.62)	89 (30.48)	0.578	0.325, 1.029	0.06
Prednisone within 30 days of index date	51 (52.58)	107 (36.64)	1.921	1.206, 3.058	<0.01
Prednisone 6 mo. prior to index date	56 (57.73)	144 (49.32)	1.447	0.898, 2.334	0.13
Azathioprine within 30 days of index date	3 (3.09)	4 (1.37)	2.250	0.504, 10.053	0.29
Leflunomide within 30 days of index date	7 (7.22)	25 (8.56)	0.819	0.345, 1.940	0.65
Hydroxychlorquine within 30 days of index date	23 (23.71)	84 (28.77)	0.784	0.462, 1.330	0.36
Sulfasalazine within 30 days of index date	6 (6.19)	38 (13.01)	0.454	0.187, 1.104	0.08
Antibiotic use 90 days prior to index date	86 (88.66)	51 (17.47)	44.984	16.41, 123.28	<0.01
**Concomitant Inflammatory Diseases**					
Psoriasis	10 (10.31)	33 (11.30)	0.892	0.411, 1.934	0.77
Crohns^†^	3 (3.09)	3 (3.09)	2.805	0. 564, 13.962	0.21
Ulcerative Colitis^†^	3 (3.09)	4 (1.37)	2.121	0.473, 9.514	0.33
Ankylosing Spondylitis^†^	4 (4.12)	7 (2.40)	1.714	0.502, 5.86	0.39
Uveitis^†^	2 (2.06)	0 (0.00)	0.138	- ∞, 0.573	0.13
**Co-morbidities**					
Diabetes mellitus	36 (37.11)	53 (18.15)	2.514	1.528, 4.134	<0.01
Chronic Kidney Disease	15 (15.46)	11 (3.77)	4.558	1.981, 10.487	<0.01
Neoplasm	9 (9.28)	12 (4.11)	2.464	0.993, 6.116	0.50
Chronic Bronchitis	44 (45.36)	81 (27.74)	2.133	1.327, 3.429	0.01
Gastroesophageal Reflux	36 (37.11)	94 (32.19)	1.282	0.777, 2.114	0.33
HIV^†^	0 (0.00)	1 (0.34)	0.333	0.009, ∞	1.00
Prior History of Skin Infection (SSSTI)	26 (26.80)	17 (5.82)	5.729	2.870, 11.434	<0.01
Discharge 3 Months Prior	14 (14.43)	20 (6.85)	2.245	1.098, 4.590	0.03
Chronic Liver Disease^†^	2 (2.06)	0 (0.00)	0.167	-∞, 0.7732	0.17
**Other Bacterial Infections**					
Pneumonia^†^	4 (4.12)	1 (0.34)	12	1.341, 107.63	0.03
Septic Arthritis^†^	1 (1.03)	1 (1.03)	.333	0.004, 26.166	0.89

^†^Cell count less than 5, Exact Analysis Used.

Multivariate models were built to examine the association between anti-TNF drug use and SSSTI, results of the final model are presented in Table 2. Based on conditional logistic regression, anti-TNF use was not significantly associated with SSSTI (OR 1.1, 95% CI: 0.61-2.03, p = 0.92) but patients with diabetes mellitus (OR 2.5, 95% CI: 1.53-4.13, p < 0.01) or a history of skin infection (OR 5.7, 95% CI: 2.87-11.43, p < 0.01) were more likely to have SSSTI.

**Table 2:** Results of the final multivariate model of patient drug exposures and comorbidities and their association with hospitalized SSSTI

**Variable**	**Case**	**Control**	**Odds Ratio**	**95%**	**Adjusted p value**
	**N = 97**	**N = 291**		**Confidence ** **Interval**	
	**Frequency (Percent)**	**Frequency (Percent)**			
Anti-TNF drug exposure	19 (19.59)	53 (18.15)	1.107	0.604, 2.028	0.92
**Diabetes mellitus**	36 (37.11)	53 (18.15)	2.514	1.528, 4.134	**<0.01**
**Prior SSSTI**	26 (26.80)	17 (5.82)	5.729	2.870, 11.434	**<0.01**

Multivariate adjustment for RA severity, diabetes mellitus, chronic kidney disease, chronic bronchitis, prior history of skin infection, pneumonia, discharge 3 months prior, prednisone use within 30 days of the index date and antibiotic use 90 days prior to the index. Age and race were also included in the initial multivariate model to assess potential confounding.

Eighty-three patients died during the study (39% cases and 15% controls, OR 3.5, 95% CI: 2.03, 6.11, p <0.01). A similar proportion of cases and controls had diabetes mellitus and of the cases who died, 36.8% had diabetes mellitus compared to 37.3% of cases who did not die. However, cases who died were more likely to have prior history of skin infection (40%) than cases who did not die (19%) during the study period (OR 2.8, 95% CI: 1.13-7.64, p = .02).

## Discussion

We studied a large US Veterans cohort of RA patients to evaluate whether SSSTI are associated with the use of anti-TNF therapies. We found SSSTI cases were no more likely to be recently exposed to anti-TNF therapies than matched controls. However, we found strong associations between diabetes and SSSTIs, as well as SSSTI and subsequent mortality independent of DMARD therapy.

Based on previous studies in other patient populations, we estimated that our study would reveal at least a two-fold greater risk of serious skin and soft tissue infections among VA hospital rheumatoid arthritis patients receiving anti - TNF drug therapy, yet we saw no significant association between anti- TNF therapy and increased SSSTI risk.

In relation to existing research, our findings differ from earlier studies that reported an association between anti-TNF drug treatment and an increase in all serious infections in RA patients [19,20]. Conversely, recent studies reported similar results to ours, and found no association between anti-TNF therapy and increased risk of infection in RA patients [22]. Another large retrospective study conducted among elderly RA patients, using data from 1995 to 2003, also did not find an increase in serious infections when comparing patients receiving anti-TNF therapy to those receiving methotrexate [26].

One of the most recent studies was conducted in a U.S. VA patient population and evaluated patient data up to 2005. In our study we analyzed data from a broader range of years in order to capture information from the period prior to 2004 (when anti-TNF drugs therapy became wide spread in the U.S.), as well as data up to 2008 (four years after anti-TNF drugs prescription was common) [27] and to enrich the number of cases of patients exposed and not exposed to anti-TNF.

Demographic and patient characteristics unique to the VA hospital population (being predominantly white, male, and older, and having a high number of comorbid conditions), as well as other limitations of the study design, including the small sample size, may be contributing factors to the difference in results between previous studies and ours.

The unique characteristics of this patient population may also limit the ability to generalize our results to patients outside the VA hospital network. In the VA cohort, approximately 2 percent of the RA patients were identified as having had a SSSTI. The prevalence of SSSTI we identified was lower than rates reported by other studies of anti-TNF drug use and all serious infections in RA patients, however, many of the studies evaluated all skin infections, rather than limiting to serious infections that result in hospitalization. Additionally, data for patients receiving acute care outside of the VISN20 for SSSTI were not captured by our study, which may account for the lower percentage of identified cases.

It is important to note that in the VA patient population studied, anti- TNF drugs were prescribed less than 20 percent of the time, while in the general population as many as 40 percent of rheumatoid arthritis patients receive anti-TNF drug treatment [17]. The prescribing patterns for anti-TNF treatment may be influenced by the high rates of co-morbidities present among these patients and by VA prescribing guidelines. In this VA patient population, average anti-TNF drug use over the entire study period was 16.7 percent for cases and 17.5 percent for controls.

Lastly, other studies have reported a significant association with prednisone use as a risk factor for infection leading to hospitalization [21,28]. In our multivariate analysis, prednisone use was not statistically significant; however, our study only evaluated the risk for SSSTI, as opposed to evaluating all major types of infection, and this, as well as the small sample size or possible dose–response relationships may have limited our power to detect an effect.

Although we observed no association between anti-TNF or other immunosuppressant therapy and SSSTI, we did identify hospitalized SSSTI as a strong risk factor for death during the study time-period. An average of 29 percent of cases died within 1 year of the index date compared to 10 percent of controls.

The higher proportion of cases who died than controls may be a marker for sicker patients with higher rates of co-morbidities. Attention was paid to potential confounding variables that may be indicators of severity of disease and increased risk of infection, such as the number of RA office visits in the year prior to infection, and the need for prednisone use, all of which are factors that may contribute to confounding by indication since sicker patients (those more likely to get infections) are less likely to be treated with these drugs. We attempted to control for this by using proxies for RA severity, but it is possible that we did not fully control for it.

Despite intrinsic limitations to our case–control study design, we attempted to reduce bias and confounding in several ways. We used validated ICD 9 codes with proven high positive predictive values for disease to identify cases of SSSTI, which may reduce misclassification bias [23], and matched cases to three sets of comparable controls to mitigate the impact of potential confounders. To minimize the risk of confounding by indication, we analyzed as indicators of disease severity the erythrocyte sedimentation rate, the number of RA clinic visits, and the need for prednisone use, however, propensity scores for severity of RA for this population were not available since the VA hospital network does not systematically collect this data. Although our RA cohort size was reasonably large, a fairly small number of SSSTI cases were identified resulting in a relatively small sample size for our case–control analysis.

In this patient cohort, significant predictors of serious skin and soft tissue infections included diabetes mellitus and having a prior history of skin infection. The impact of these findings suggests that patients in the VA cohort with a history of prior skin infection or diabetes mellitus are at elevated risk for serious skin and soft tissue infections and hospitalization. The increased mortality among cases indicates that intervention and careful monitoring of these patients may be important.

## Conclusion

The findings of our research on the risk of serious skin and soft tissue infection in this patient group highlights the need for further study to provide insight into improving clinical care and preventing future complications and morbidity in this patient population.

SSSTI were significantly associated with diabetes mellitus and history of skin infection but not associated with anti-TNF use in the VISN 20 RA patient cohort. However, patients with SSSTI were much more likely to die than matched controls. These findings highlight the need to better understand trends in diabetes and SSSTI cares among US veteran’s and argue for further evaluation of specific co-morbidities and other factors that could be associated with SSSTI related mortality.

## Abbreviations

Anti-TNF: Anti-tumor necrosis factor alpha; CI: Confidence Interval; DMARD: Disease-modifying anti-rheumatic therapy; NSAID: Non-steroidal anti-inflammatory drug; OR: Odds Ratio; SSSTI: Serious skin and soft tissue infections; US: United States; VA: Veterans Administration; VISN 20: Veterans Integrated Service Network 20.

## Competing interests

The authors declare that they have no competing interests.

## Authors’ contributions

NW designed the study, conducted the statistical analysis, and drafted the manuscript. PS provided rheumatoid arthritis clinical expertise and assisted with drafting the manuscript. RF provided statistical assistance and helped draft the manuscript. CV assisted with the coordination of the study, review of the manuscript, and the statistical analysis. KW conceived of the study, and participated in its design and coordination and helped to draft the manuscript. All authors read and approved the final manuscript.

## Pre-publication history

The pre-publication history for this paper can be accessed here:

http://www.biomedcentral.com/1471-2334/13/533/prepub
